# Spatial Accuracy and Variability in Dart Throwing in Children with Developmental Coordination Disorder and the Relationship with Ball Skill Items

**DOI:** 10.3390/ejihpe14040067

**Published:** 2024-04-16

**Authors:** Faiçal Farhat, Achraf Ammar, Nourhen Mezghani, Mohamed Moncef Kammoun, Khaled Trabelsi, Haitham Jahrami, Adnene Gharbi, Lassad Sallemi, Haithem Rebai, Wassim Moalla, Bouwien Smits-Engelsman

**Affiliations:** 1 Research Laboratory: Education, Motricity, Sport and Health, EM2S, LR19JS01, High Institute of Sport and Physical Education of Sfax, University of Sfax, Sfax 3000, Tunisia; faical.farhat@isseps.usf.tn (F.F.); moncef.kammoun@isseps.usf.tn (M.M.K.); khaled.trabelsi@isseps.usf.tn (K.T.); lassadsallemi@gmail.com (L.S.); wassim.moalla@isseps.usf.tn (W.M.); 2 High Institute of Sport and Physical Education of Sfax, University of Sfax, Sfax 3000, Tunisia; adnenegharbi@yahoo.fr (A.G.); haithem.rebai@isseps.usf.tn (H.R.); 3 Department of Training and Movement Science, Institute of Sport Science, Johannes-Gutenberg-University Mainz, 55122 Mainz, Germany; 4 Research Laboratory, Molecular Bases of Human Pathology, LR19ES13, Faculty of Medicine of Sfax, University of Sfax, Sfax 3000, Tunisia; 5 Interdisciplinary Laboratory in Neurosciences, Physiology and Psychology: Physical Activity, Health and Learning (LINP2), UFR STAPS, UPL, Paris Nanterre University, 92000 Nanterre, France; 6 Department of Sport Sciences, College of Education, Taif University, Taif 21974, Saudi Arabia; nsmezghanni@tu.edu.sa; 7 College of Medicine and Medical Science, Arabian Gulf University, Manama 293, Bahrain; hjahrami@health.gov.bh; 8 Physical Activity, Sport and Health Research Unit, National Observatory of Sport, Tunis 1003, Tunisia; 9 Sports Performance Optimization Research Laboratory (LR09SEP01), National Center for Sports Medicine and Science (CNMSS), Tunis 1003, Tunisia; 10 Physical Activity, Sport and Recreation, Faculty Health Sciences, North-West University, Potchefstroom 2520, South Africa; bouwiensmits@hotmail.com; 11 Department of Health and Rehabilitation Sciences, University of Cape Town, Cape Town 7925, South Africa

**Keywords:** children, developmental coordination disorder, motor skill-related fitness, ball skills, dart throw, task difficulty

## Abstract

The present study aimed to examine precision and variability in dart throwing performance and the relationships between these outcomes and bouncing, throwing and catching tasks in children with and without DCD. Children between the ages of 8 and 10 years (n = 165) were classified according to results obtained on the Movement Assessment Battery for Children (MABC-2) and divided into three groups: 65 children with severe DCD (s-DCD), 45 with moderate DCD (m-DCD) and 55 typically developing children (TD). All children performed the dart throwing test and the ball skill items of the Performance and Fitness Test (PERF-FIT). The accuracy and variability of dart throwing tasks were significantly different between TD and s-DCD (*p* < 0.01), and also between m-DCD and s-DCD (*p* < 0.01). Participants with s-DCD were also found to perform significantly worse on all PERF-FIT ball skill items than m-DCD (*p* < 0.001), and m-DCD were significantly poorer than TD (*p* < 0.001). The dart score and coefficient of variation of the long-distance task appear to be significant predictors for the ball skills and explain between 24 to 29% of their variance. In conclusion, poor results in aiming tasks using darts in children with DCD corroborate with the explanation of deficits in predictive control since the tasks require ballistic movements.

## 1. Introduction

Children with developmental coordination disorder (DCD) exhibit slow, effortful, imprecise, and ill-coordinated movements and are more depended on visual information [1]. The leading hypothesis that best explains motor coordination and skill learning deficits is that children with DCD have difficulties with predictive motor control [2]. In this context, studies examining predictive motor control, such as adaptations in grip force, anticipatory postural adjustments, and predictive control of eye movements, have shown that motor outcome prediction is impaired in children with DCD [2]. In addition to children with DCD having less accurate, slower, and more variable motor performance [3], they also have difficulties visually tracking moving objects [4], which is particularly important in active playground games using balls, such as catching a ball or aiming [2].

The deficits in predictive control in children with DCD can manifest as problems with fine and gross motor skills such as throwing and catching a ball, a set of skills that children with DCD have considerable difficulty with [5]. Proficient ball skills are essential because playground games and physical education classes often include aiming, throwing and catching activities [6]. Indeed, problems with coordination in children with DCD often restrict them from executing functional skills, which are needed for effective participation in sports and leisure activities [7,8]. These difficulties with motor control during ball catching lead to a large number of aiming and catching errors [9]. 

Motor control theories state that when an action is planned, the motor parameters related to that action such as the trajectory, speed, and required precision are represented as an internal model [10]. Internal models contribute to efficient, accurate and smooth motor performance, limiting the requirement for the motor system to depend on slower varieties of feedback-based control to correct the movement [11]. This ability permits children to anticipate movement results, determine the required control functions and achieve desired outcomes such as distance, timing and force [12]. Children with DCD have a limited ability to utilize internal models for motor control [13]. Jucaite et al. [14] reported that postural and manual forces could not be scaled in either the temporal or amplitude domains in children with DCD. Their study demonstrated that children with DCD modified their grip forces but exhibited temporal delays in the corresponding postural adaptations. Impaired force control was shown to be related to timing [15] and fine-tuning of the force [16]. In this context, Smits-Engelsman et al. [17] also showed that children with DCD produced more variable force trajectories than controls did. 

In clinical practice, ball skills are typically measured using tests such as M-ABC [18] and BOT [19]. Standardized tests assess ball skills in a very predictable context, which makes the tests reliable but more distant from real-world ball games where trajectory prediction is one of the determining factors [20]. The spatial and temporal accuracy of the movement patterns are crucial because the hands (thus the body) must catch and throw the ball at the right time and place, or release a ball or dart with the right precision. This requires motor control, which refers to an adaptive process of the variables given the motor pattern, such as force, speed, and timing [4]. The recently developed Performance and Fitness test (PERF-FIT) contains repetitive bouncing and catching, throwing and catching items, where children can move freely around and adapt their body position when needed based on available feedback [21]. 

A task in which fine-tuning of a skilled open-loop-controlled motor action can be tested is throwing darts. For throwing movements including darts, control is related to both the target distance and mass of the object manipulated. Likewise, in the current study, dart throwing requires more end point precision than bouncing and catching, throwing and catching, or a throw for distance (explosive power). On the other hand, less precise executed bouncing or throwing movements could still be corrected for if hand and body movements are adapted fast enough to the throwing error so the ball can still be caught, while error correction is not possible once the dart has been released from the hand. Hence, these tasks have both similarities and differences from a motor control perspective. 

Many aspects of motor control and executive function determine the success of an aiming movement. Darts are thrown overarm, with forward movement of the arm, mainly elbow extension, but this also requires an adequate throwing position. The adjustment of the movement properties (direction and velocity) and the temporal accuracy of the grip opening during the hand’s forward movement to release the dart will regulate where it lands. Moreover, these parameters need to be adapted with respect to the external frame of reference defined by the target board. Hence, dart throws include, in addition to the ability to process information to create the right movement pattern, the ability to grade forces with extreme precision [22]. Thus, when throwing a dart or a ball, the online configuration of several joints (i.e., shoulder, elbow, and wrist) must be monitored during the movement to identify when the arm is in the right position to release the object.

In the present study, we investigated dart throwing as a typical example of an open-loop (ballistic) aiming task, hence requiring predictive control. Predictive control is based on prior knowledge of the dynamics of moving arms and the internal disturbances caused by arm movements upon the body. The anticipated variables (such as force, timing, and changes in visual input) are based on acquired associations between output signals and their effects on effectors during repeated practice or experience [23]. Based on this knowledge, the central nervous system can correctly program anticipatory postural adaptations [24]. Most studies comparing movement accuracy in children with DCD have used tasks where visual correction during movement execution is possible. For instance, it has been found that when children with DCD move toward a target in visually guided aiming movements, they make considerable endpoint errors and have less fluent movement profiles than their well-coordinated peers [16]. Importantly, in these visually guided aiming movements, children may choose to move more slowly to be more accurate, which is not an option in dart throwing. However, vision still plays an important role in a dart throw task. To hit a specified target with a dart, movement parameters, which are determined in a body-related frame of reference, must be calibrated relative to an external frame of reference. Generally, vision establishes the link between these various points of reference [25].

To our knowledge, no empirical study has investigated the differences between children with DCD and TD children in terms of throwing darts and ball skills. In these skills, children must manipulate different objects (darts and balls) with different strategies under various motor control conditions. Therefore, the purpose of the present study was to examine the precision and variability of dart-throwing performance and the relationships between these outcomes and ball skills, which have aiming and catching components, in children with and without DCD.

Based on previous studies, we hypothesized that, compared with TD children, children with DCD would be less accurate (as measured by the dart score) and have greater variability (as measured by the coefficient of variation or CV) in performing a dart task. We also expected that this difference would be greater in the more difficult version of the task (standing further away from the target) and greater in the severe DCD group than in the moderate DCD group and TD children. Moreover, moderate correlations are expected between the dart outcomes and the ball skill-related activities of the PERF-FIT because they only partly share a common underlying construct (aiming or throwing at a target) and control modes (predictive versus online control).

## 2. Materials and Methods

### 2.1. Procedure

The protocol and study design were approved by the Ethical Committee of the University Hospital of Sfax, Tunisia (CPP SUD N° 0301/2021). Children were tested at their schools. After a full explanation of the procedure and prior to testing, all the parents provided written consent and signed child assent forms. Twelve assessors received 10 h training on all the outcome measures. Children’s length and weight were measured before testing. All tests were administered at a maximum of one week apart. Administration of the MABC-2 took approximately 25–35 min. The PERF-FIT required approximately 25–45 min. The dart throwing test took 15–25 min. Before scoring, each test item was explained to the child, demonstrated, and practiced according to the manuals.

### 2.2. Participants

To select the participants, teachers and parents were asked to identify children with motor coordination problems based on their observations on the playground, in class or at home. Children with DCD were classified using the four DSM-5 criteria [26]. All the children aged 7 to 10 years with an MABC-2 score at or below the 16th percentile (Criterion A); identified by a parent or teacher as having a motor coordination impairment (Criterion B); whose parents noted difficulty throughout the early developmental period (Criterion C); where no medical condition or comorbidity known to interfere with motor abilities was noted; and whose teacher confirmed the absence of intellectual or cognitive disability (Criterion D) appeared to meet the DCD criteria. Through this procedure, 110 children with DCD were selected to participate in this study. These children were age- and sex-matched at a 2:1 ratio, with 55 TD children from the same grade. TD children were recruited from the same classes in the school as the children with DCD were. No additional developmental conditions such as attention deficit hyperactivity disorder, dyslexia, or autism spectrum disorder were reported in any of the groups.

The inclusion criteria for the TD children were as follows: (1) no evidence of functional motor problems as observed by their teacher or parent, (2) a score above the 16th percentile on the MABC-2, (3) no diagnosis of a significant medical condition as reported by a parent and (4) the absence of intellectual or cognitive impairment as confirmed by their teacher. Based on their MABC-2 scores, the children with DCD were divided into moderate DCD (n = 45) and severe DCD (n = 65) groups to examine the impact of DCD on the severity of motor deficiencies.

### 2.3. The Movement Assessment Battery for Children-2 (MABC-2)

All the children completed the MABC-2 age band 2 (7- to 10-year-old children). The MABC-2 test was used to measure motor coordination [18] and to confirm DSM-5 Criterion A for DCD. The MABC-2 test consists of eight items that are evaluated on three different components: manual dexterity, aiming and catching, and balance. A percentile score of five or less indicates severe motor problems (we will refer to this group as severe DCD or s-DCD), while a score between 9 and 16 suggests that the child is at risk of having movement difficulties (we will refer to this group as moderate DCD or m-DCD), and a score > 16th percentile indicates normal motor performance. The MABC-2 test has demonstrated good validity and test–retest reliability, with ICC values ranging from 0.92 to 0.98 [26].

### 2.4. Performance and Fitness Test (PERF-FIT)

The PERF-FIT is a functional measure of motor skill-related fitness in children [27]. The PERF-FIT is the first standardized test to establish norms for African children and is an affordable testing tool for low-resource areas. The test is suitable for this age group (elementary school children). The items of the PERF-FIT were designed to be used throughout the full age range. The PERF-FIT has good structural and ecological validity, excellent content validity, and good reliability [28,29].

The PERF-FIT consists of two subscales. The power and agility subscale comprise three agility items (running, stepping, and side jumping) and two explosive power items (overhead throw and standing long jump). The motor skills performance subscale contains five series of tasks with increasing difficulty: (1) bounce and catch ball (2), throw and catch ball, (3) static balance and dynamic balance, (4) jumping, and (5) hopping. For this study, only the ball skills items and the explosive power overhead throw were used because of their expected relationship with the dart task and M-ABC. In an earlier study, throwing and bouncing item series of the PERP-FIT were low to moderately related to the MABC-2 aiming and catching score [21].

### 2.5. Dart-Throwing Test

The dart throwing test started after the initial training period. This training period included 15 darts thrown in five blocks of three from two distances (2.37 and 3.56 m) [30]. The task was to throw the darts as close to the bullseye as possible. The two distances were marked by a line on the floor. The test runs consisted of six throws at each of the two distances, in a randomized order; the height of the official dartboard was placed on a wall so that its center was at eye level for each child. The children were required to maintain the same throwing technique in both conditions.

#### Score Calculations

The throw was scored depending on its position on the board (0–10). A dart that missed or bounced off the board received a score of zero. The target consisted of a series of 10 concentric rings. Two outcomes were used to measure the accuracy and consistency [31]. The first was the mean score of the six throws. This score can range from zero (all misses) to ten (all bullseye); it can be considered a measure of accuracy, with a high score indicating high accuracy. The second measure of performance was the coefficients of variation (CV) of the score: SD score/mean score, a lower coefficient indicating a higher consistency.

### 2.6. Statistical Procedure

All variables were examined to determine whether the distributions were normal or skewed. No outliers were present in the data. ANOVA was used to test for differences in demographic variables among the three groups. The chi^2^ test was used to determine the sex distribution across groups.

Repeated measures ANOVA were used to examine the effect of tasks (within subject: short and long distance) and group (between subject: TD, m-DCD, s-DCD) and possible interactions. One-way ANOVA was used to examine differences between the three groups on the PERF-FIT measures. A post hoc test with Bonferroni correction was used if main effects for group or interactions were found. The magnitude of the differences per group was determined using Cohen’s d-values of 0.5 (moderate effect size) and 0.8 (large effect size) [32].

To test for task specificity of throwing skills, Pearson’s correlations were calculated to assess the relationships between MABC-2 aiming and catching scores, PERF-FIT ball skill items (raw scores) and dart throw performance. A stepwise multiple regression analysis was used to determine the best set of predictor variables (dart outcomes) for the ball skill items.

The significance level was set at *p* < 0.05. All the statistical analyses were conducted using the Statistical Package for the Social Sciences software (SPSS, version 28.0; SPSS, Inc., Chicago, IL, USA).

## 3. Results

### 3.1. Participants

The anthropometric data of the two DCD groups and the control group are shown in Table 1. Significant differences in weight and body mass index were found between groups (*p* < 0.001). Post hoc tests showed that the TD group was different from the DCD group, but the m-DCD and s-DCD groups were not different in weight or BMI. There were no significant group differences in age or height (*p* > 0.05). Differences in the total and subscale scores on the MABC 2 between the groups are also shown in Table 1. The sex distributions of the three groups were comparable (Chi^2^ 1.19, *p* = 0.55) (Table 2).

### 3.2. Dart Throw Measurements

Dart Scores: Table 3 reports the means and standard deviations for the TD and the DCD groups on dart scores and effect sizes. 

**Figure 1:**
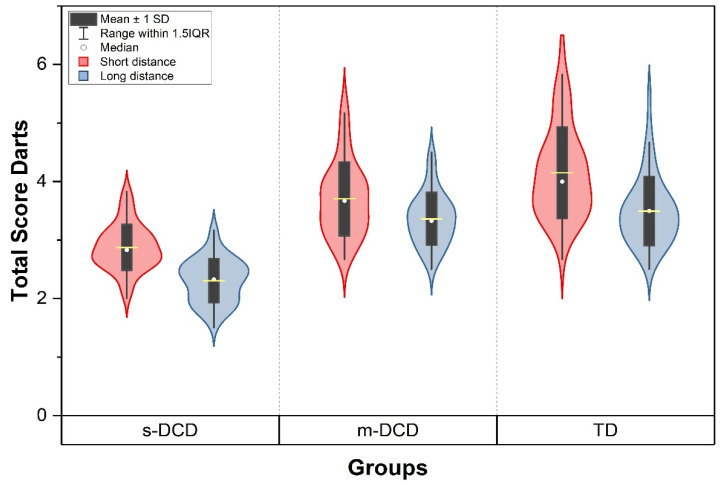
Dart score in the two conditions for the three groups. Scores are lower in the long-distance task compared to the short distance. The s-DCD group is poorest in the task. For the short-distance score, differences between TD and s-DCD, TD and m-DCD, and m-DCD and s-DCD are significant (*p* < 0.001). For the long-distance performance, the dart score is not different between TD and m-DCD. Differences between TD and s-DCD, and m-DCD and s-DCD are significant (*p* < 0.001). s-DCD = severe DCD, m-DCD = moderate DCD, TD = typically developing.

A repeated measures ANOVA revealed a significant main effect of group for dart scores (F_(2, 162)_ = 95.29, *p* < 0.001; Figure 1). Additionally, a large main effect of task was found, showing that the 50% greater distance led to darts landing less close to the target (F 1,162 = 275.91, *p* < 0.001). Importantly, the statistical analysis revealed a significant interaction effect between group and task for dart score (F _(2, 162)_ = 8.05, *p* < 0.001). Indicating that the groups responded differently in the task conditions. 

Post hoc analysis revealed that for the short-distance comparisons, dart score was significantly different between the TD- and m-DCD, TD and s-DCD, and m-DCD and s-DCD (all *p* ≤ 0.001). However, for long distance, the dart score was not different between the TD and m-DCD groups (*p* = 0.52) but only for the comparisons between TD and s-DCD (*p* < 0.001), and m-DCD and s-DCD (*p* < 0.001). 

**Figure 2:**
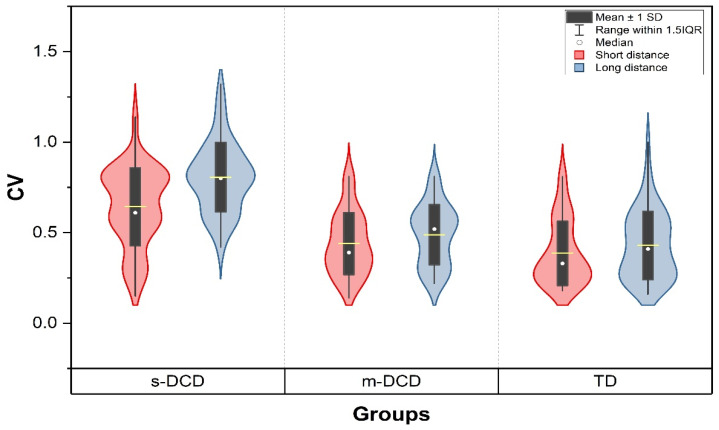
Coefficient of variation in the two conditions for the three groups. Performance is more variable in the long-distance task. Children with s-DCD are poorest in the task. CV in not different between TD and m-DCD groups. But differences in variability between TD and s-DCD, and m-DCD and s-DCD are significant (*p* < 0.001). s-DCD = severe DCD, m-DCD = moderate DCD, TD = typically developing, CV = coefficient of variation.

Coefficient of variation: Table 4 reports the means and standard deviations for the TD and the DCD groups on CV and effect sizes. A repeated measures ANOVA revealed a significant main effect of group for variability (F _(2, 162)_ = 56.18, *p* < 0.001) (Figure 2). Additionally, a large main effect of task was found, showing that the greater distance led to more variability (F _(1, 162)_ = 55,16, *p* < 0.001).

Moreover, the statistical analysis revealed significant interaction effects between group and task for variability (F _(2, 162)_ = 13.25, *p* < 0.001). Post hoc analysis of the CV showed that TD and m-DCD did not differ either for the short- (*p* = 0.52) or for the long-distance tasks (*p* = 0.33). The other post hoc comparisons for CV between TD and s-DCD and m-DCD and s-DCD were significantly different (*p* < 0.001).

### 3.3. The PERF-FIT Measures

**Figure 3:**
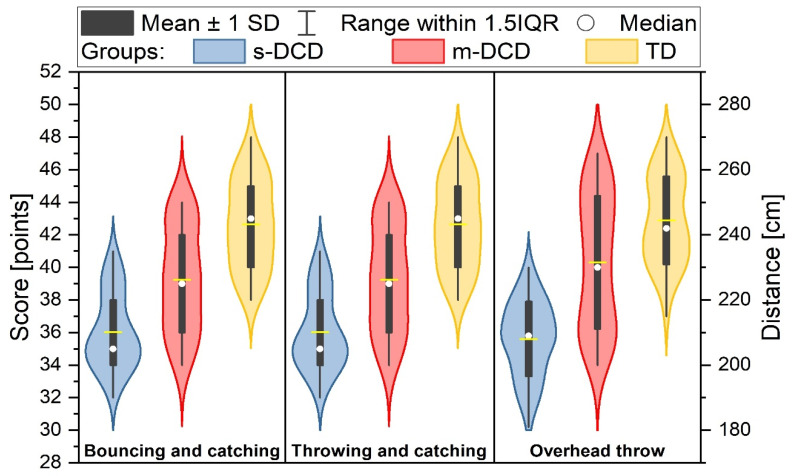
Ball skills items for the three groups. Children with s-DCD are poorest at bouncing and catching, throwing and catching, and overhead throw. Left scale for bouncing and catching and throwing and catching. Right scale for overhead throw. Scores are different between TD and m-DCD, TD and s-DCD and also between s-DCD and m-DCD group (*p* < 0.001). s-DCD = severe DCD, m-DCD = moderate DCD, TD = typically developing.

Significant differences in upper extremity muscle power, throwing and catching, and bouncing and catching were found between the groups (Table 5). Post hoc analysis also revealed that the three groups differed from each other in terms of these skills (*p* < 0.001). The children with s-DCD showed significantly lower scores than the m-DCD children did, and the m-DCD children had significantly poorer scores on all items than the TD children did; see Table 5 and Figure 3.

### 3.4. Associations between Dart Performance and Ball-Skill Items

**Figure 4:**
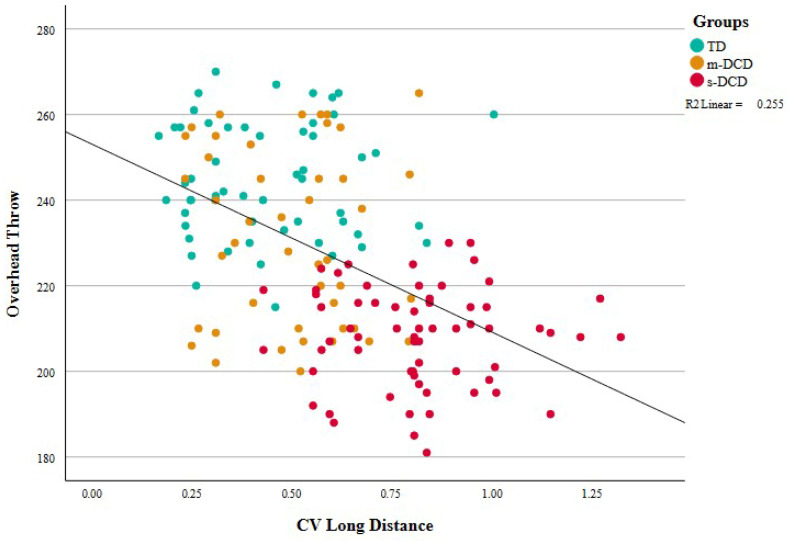
Scatterplot showing the association between the coefficient of variation of the dart score in the long-distance task and the distance thrown in the overhead throw of the sandbag (explosive power item of the PERF-FIT). Larger variability coincides with shorter distance of the throw, leading to negative correlations. TD = typically developing, m-DCD = moderate DCD, s-DCD = severe DCD. CV = coefficient of variation.

**Figure 5:**
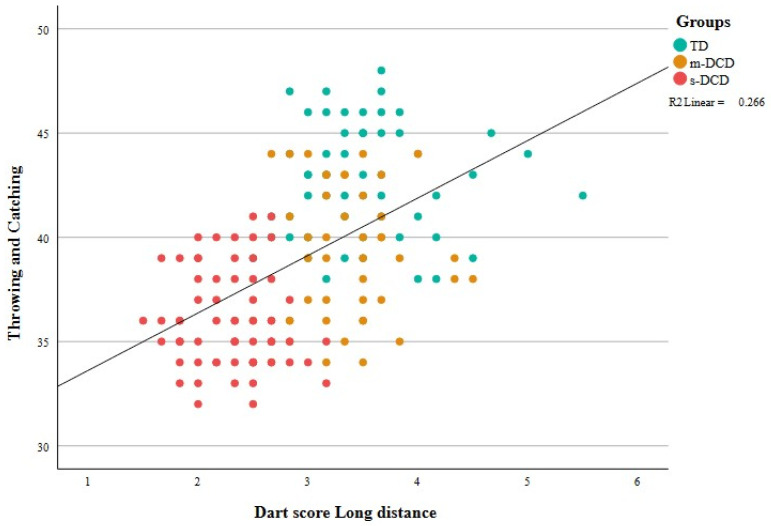
Scatterplot showing the association between the dart score in the long-distance task and the number of points in the throwing and catching task of the PERF-FIT. More precision of the dart throws coincides with more caught balls in the throwing and catching task, leading to positive correlations. TD = typically developing, m-DCD = moderate DCD, s-DCD = severe DCD.

Moderate correlations were found between dart outcomes and ball skill outcomes. See Table 6 and Figure 4 and Figure 5. High correlations for MABC aiming and catching scores were expected because these scores are part of the selection criteria for the classification of children into groups. For the other ball skills, associations with dart outcomes are in the comparable range (0.35–0.50) and are slightly greater for long-distance outcomes. The association between explosive power and dart outcomes is remarkable because the two tasks have very different accuracy constraints. Results indicate that children who can generate more power in the PERF-FIT overhead throw test also exhibit less variability in dart throws. Aiming a bean bag at a circle on the mat and aiming at the dart board were also moderately correlated.

Multiple regression (stepwise) was used to find the best set of predictors for the ball skill-related items. In the first step of the analysis, the regression analysis showed that BMI is not a significant predictor of dependent variables such as aiming, bouncing and throwing. Therefore, we did not enter BMI as a possible predictor variable in the reported models. The results are summarized in Table 7. In the first model, all four dart outcomes were entered as predictors. The dart scores and CV of the short-distance task did not significantly predict the outcomes of any of the ball skill-related items; therefore, they were not included in Model 2. The significance probability of Model 2 with only long-distance outcomes was significant (*p* < 0.001), indicating that the regression model was suitable. Furthermore, multicollinearity tests demonstrated that all the indicators had variance inflation factor (VIF) values less than three, demonstrating that the explanatory variables in Model 2 were not highly linearly related. Throwing and bouncing outcomes were best predicted by accuracy in the long-distance task (dart score), and the CV, as the second predictor, added only a small percentage. For the MABC-2 items aiming and catching a beanbag, variability in the dart results (CV) was the best predictor, and the darts score added only approximately 2%. The same pattern was observed for the overhead throws.

## 4. Discussion

Aiming a ball at a target or other person so that it can be caught is a task that includes components that are common in many leisure and sports activities. Children with DCD are known to have poor aiming and throwing skills. The causes of the less coordinated movements in children with DCD are still unclear; only few studies have investigated ballistic movement precision to an external focus point [33,34,35], in which the central nervous system must predict the force and the final position of an object at which it must be released (predictive control). As hypothesized, compared to TD children, children with s-DCD were less accurate (as measured by the dart score) and had greater variability (as measured by the coefficient of variation) in performing a dart task. Children with m-DCD were only less accurate in the short-distance task. It was also confirmed that the task is harder if the children are standing further away from the target. Importantly, children with severe and more moderate coordination deficits differed in terms of the tested tasks, such as precision throwing (dart) or generating explosive power (overhead throw with a heavy sandbag). Not many studies looked at differences in performance between children with moderate and severe DCD. Given that the effect sizes of the differences between m-DCD and s-DCD indicate large differences (Cohen’s d are larger than 1) on all dart and ball skill items, this signifies that the cutoff values used for classifying DCD will impact the degree of task performance on other tasks.

Moderate correlations were found between the dart outcomes and the ball skill-related activities. The accuracy and variability of the long-distance dart throws explained approximately 25% of the variability in the ball skill-related activities. Both the correlations and the regression results indicated that the long-distance task, in which a small initial directional mistake will have large influence on the endpoint of the dart, best explain the variance in the ball skills.

### 4.1. DCD and Aiming

Converging findings indicate that children with DCD make more errors when performing goal-directed movements [36,37,38]. In our study, children with s-DCD also showed less accurate performance in ballistic movements. Whether and where a dart will hit the target depend on the combination of position, speed and direction of motion at the moment that it is released from the hand. According to the current study, the performance of children with s-DCD was significantly poorer for each dart outcome than that of their healthy peers. Children with m-DCD only differed from TD in the number of times they were able to hit the target in the short distance task but did not show more variability than their healthy peers.

Aiming with a dart at a target is clearly different from reaching for a target or object. It has been hypothesized that children with DCD apply compensatory strategies to overcome difficulties when reaching for an object by changing the initiation of the reaching phase and grasping phase by a higher degree of both the intra- and interlimb coupling and fixating the joints [39], as stiffness increases the ease of control [23]. The extra challenge in aiming is that online correction of the aiming trajectory (dart in flight) is not possible, which increases the sensitivity to end point errors. The ability to estimate the dynamic position of the joints involved in the throw is critical for the timing and control of the dart. Children with s-DCD may have been less able to properly sequence joint rotations, as they are known to have deficits in kinesthetic information processing [40] and, to some extent, difficulties integrating perceptual information into action.

The fact that ample explosive power needs to be generated for the dart to cover the distance to the board can also be concluded based on the relationship with overhead throw (r = 0.49), as the distance covered was significantly lower in children with DCD (mean 208 cm for s-DCD and 245 cm for TD). This finding is consistent with previous studies that indicated a significantly lower performance in upper limb power in children with DCD [41,42,43] and a moderate relation (r = 0.50), between overhead throw and distance thrown with a tennis ball [21].

In addition, compared to TD children, children with s-DCD had not only a less accurate performance but also had a greater coefficient of variation. Previous research reported children with DCD to be less accurate and more variable in their trial-to-trial performance than their typically developing peers, regardless of the motor-based activity under scrutiny [44,45,46]. Several studies have attributed this variability to different sources of neuromotor noise. Neural variability or noise is present at all stages of sensorimotor control, from sensory processing and central planning to the outputs of the motor system, leading to uncertainty about the movement endpoint. Excessive neural noise in the motor system has been associated with poor motor prediction in children with DCD [23]. Moreover, Smits-Engelsman et al. [17] showed that children with DCD generate more variable force than controls. A higher movement variability, lower movement accuracy and longer movement duration could explain the observed performance problems [47]. Due to this variability in performance, it will be much harder to distinguish statistical regularity from all sensory information, leading to impairment in so-called ‘prior’ development, which is needed for predictive control [48]. Due to inefficient predictive control, they rely heavily on feedback mechanisms and exhibit slower processing speed and inefficient preparation for movement [47].

### 4.2. DCD and Ball Skills

Our results are consistent with previous studies in which Brazilian children with DCD performed worse on the PERF-FIT ball skill items than their healthy peers [28] Findings from Schott [49] and Yu et al. [50] also pointed out their disadvantage in object control. Similarly, we found that children with DCD scored significantly lower on the modified ball catching item of the Test of Gross Motor Development, examining the movement patterns displayed, than their peers did [51], as well as on a specifically developed ball catching test [52]. In the last study, a significant correlation was found between the visual timing task and the ball catching test in children with DCD. Our study confirmed the relationship between MABC-2 and PERF-FIT ball skills with the dart throwing test, comparable to the low to moderate interrelations between different ball skills that were reported in earlier studies [41].

Children with DCD were as fast and as accurate as their peers in their initial performance of the simple task. However, they were slower and less accurate when performing complex and novel visual motor tasks [52]. This is in line with our results; the dart scores and the CV of the more difficult long-distance throws significantly predicted scores on the ball skill items, while the outcome of the easier task (short distance) did not.

Our results show that the dart scores in the more difficult condition (long distance) was not different between TD and m-DCD; only differences between TD and S-DCD and m-DCD and s-DCD were found. This result could be explained by the fact that for the typically developing children too, the 50% increase in distance from the target made it more difficult, leading to lower scores for that group (see Figure 1), diminishing the difference between TD and m-DCD. Importantly large differences were found between the three groups in all ball skill items. These findings suggest that children with m-DCD and s-DCD experience difficulties when catching a ball, where online corrections are still possible [53]. However, longer response times and deficits in visual motor integration may limit the ability of these children to do so adequately [51]. This finding is in line with findings that children with DCD also produce larger endpoint errors in visually controlled upper limb aiming movements [54], which might partly be explained by deficits in visual pursuit [55]. In accordance with these findings, Miles et al. [56] showed that quiet eye training improved the ability of children with DCD to focus on a target on the wall prior to the throw, followed by better anticipation and pursuit tracking of the ball, which in turn led to improved catching technique.

### 4.3. Practical Implications

Precision performance difficulties are found to be a defining characteristic of DCD, the presence of such a marker could be used to offer novel diagnostic avenues to be explored in the future. Ensuring that specific attention in future research is allocated to task requirements when designing experimental tasks will help to continue the exploration of precision tasks in children with poor coordination. Our results also made clear that the motor test cut off scores used will lead to studying or treating a different group of children. Our findings support that the greatest differentiation of children with DCD from their peers and between severity groups occurs when performing a complex task, like throwing and catching. Efforts to help children with DCD gradually build up from simple to complex tasks could potentially bridge the gap between their performance and that of their peers. Moreover, our findings show that predictive control as needed in ballistic movements only explains part of the ball skill performance in which online control of body and hand position is needed to correct inaccuracies in the initial movement.

### 4.4. Limitations

In children with DCD, the literature seems to indicate that there is a clear association between visual motor deficits and ball skills. No tests were included to verify this in the current study. More trials in the dart experiment might have provided information on whether the accuracy of the children would have improved if more practice was given. Future studies could also look into eye movements during the dart task. To confirm whether the present results can be explained by predictive control deficits, anticipatory modulation of muscular activity during a dart throw needs to be measured in future studies. Insights into the timing and control of the arm and eye movements are crucial for understanding the differences between children with and without DCD. The quantitative data (scores and coefficient of variation) only provide information about the outcomes and do not capture the specific actions of the shoulders, arms, and hands during the throwing process. Further kinematic studies could provide explanations regarding the relationship between these factors.

## 5. Conclusions

The present study showed deficits in aiming at a target, a ballistic movement, in children with DCD. These children with more severe DCD showed a more variable performance, which was not a feedback-based correction. Deficits were greater when the children stood further away from the target, where smaller deviations had a greater impact on the endpoint accuracy. These poor results corroborate the explanation of deficits in predictive control since the task requires ballistic movements, which are different from visual-guided goal-directed movements where online sensory feedback can be used to accurately reach the target or object after the initiation of the movement. Motor ability influenced the dart task, leading to a more pronounced effect in the s-DCD group and task-dependent differences between the m-DCD and TD groups. Notably, for all the ball skills tasks tested, the three groups were evidently different from each other.

## Figures and Tables

**Table 1:** Demographic and background information of the three groups (TD, m-DCD and s-DCD).

	s-DCD (n = 65)		m-DCD (n = 45)		TD (n = 55)		Statistics	
	Mean	SD	Mean	SD	Mean	SD	F-Value	*p*-Value
Age (years)	9.05	0.82	8.93	0.86	9.07	0.84	0.38	0.68
Height (m)	1.38	0.06	1.38	0.06	1.39	0.07	1.27	0.28
Weight (kg)	38.3	4.47	34.3	4.93	31.8	4.54	30.45	<0.001
BMI (kg m^2^)	20.03	1.18	17.62	2.95	16.25	1.25	30.96	<0.001
Total Standard Scores	3.7	0.8	6.4	0.5	9.60	1.1	711.35	<0.001
Manual Dexterity	5.0	1.2	7.4	0.9	9.4	1.0	265.19	<0.001
Aiming and Catching	5.1	1.3	7.2	0.9	10.1	1.4	252.52	<0.001
Balance	4.8	0.9	7.1	0.9	9.5	1.2	58.41	<0.001

SD = standard deviation, TD = typically developing, m-DCD = moderate DCD, s-DCD = severe DCD.

**Table 2:** Gender distribution over groups (no significant differences).

Group	n	% Boys	% Girls
TD	55	49.1	50.9
m-DCD	45	57.8	42.2
s-DCD	65	47.7	52.3
Total	165	50.9	49.1

TD = typically developing, m-DCD = moderate DCD, s-DCD = severe DCD.

**Table 3:** Means (M) ± standard deviations (SD) for the dart scores and effect sizes for the total differences (eta squared) between the 3 groups and pair-wise comparison (Cohen’s d).

	s-DCD (n = 65)		m-DCD (n = 45)		TD (n = 55)		3 Groups	TD/m-DCD	TD/s-DCD	m-DCD/s-DCD
	Mean	SD	Mean	SD	Mean	SD	Eta Squared	Cohen’s d	Cohen’s d	Cohen’s d
Dart Score Short	2.87	0.39	3.70	0.63	4.15	0.79	0.45	0.62	2.11	1.64
Dart Score Long	2.30	0.38	3.36	0.45	3.49	0.59	0.58	0.25	2.44	2.58

SD = standard deviation, TD = typically developing, m-DCD = moderate DCD, s-DCD = severe DCD.

**Table 4:** Means (M) ± standard deviations (SD) for the CV scores and effect sizes for the total differences (eta squared) between the 3 groups and pair-wise comparison (Cohen’s d).

	s-DCD (n = 65)		m-DCD (n = 45)		TD (n = 55)		3 Groups	TD/m-DCD	TD/s-DCD	m-DCD/s-DCD
	Mean	SD	Mean	SD	Mean	SD	Eta Squared	Cohen’s d	Cohen’s d	Cohen’s d
CV Short	0.64	0.22	0.44	0.17	0.39	0.18	0.27	−0.30	−1.29	−1.03
CV Long	0.81	0.19	0.49	0.17	0.43	0.19	0.47	−0.33	−1.97	−1.73

SD = standard deviation, TD = typically developing, m-DCD = moderate DCD, s-DCD = severe DCD, CV = coefficient of variation.

**Table 5:** Results of the ANOVA for PERF-FIT measures. Data are presented as means (M) ± standard deviations (SD)m F and *p*-values and effect sizes (eta squared) between the 3 groups and pair-wise comparison (Cohen’s d).

	s-DCD (n = 65)		m-DCD (n = 45)		TD (n = 55)		Statistics		3 Groups	TD/m-DCD	TD/s-DCD	m-DCD/s-DCD
	Mean	SD	Mean	SD	Mean	SD	F	*p*-Value	Eta Squared	Cohen’s d	Cohen’s d	Cohen’s d
Bouncing and Catching (#)	38.40	1.82	40.60	2.04	42.49	2.36	58.41	<0.001	0.42	0.85	1.96	1.15
Throwing and Catching (#)	36.02	2.33	39.24	3.23	42.64	2.71	88.09	<0.001	0.52	1.15	2.64	1.18
Overhead Throw (cm)	208.0	11.5	231.6	20.5	244.5	13.6	90.19	<0.001	0.53	0.76	2.92	1.49

SD = standard deviation, TD = typically developing, m-DCD = moderate DCD, s-DCD = severe DCD. # Number of balls caught.

**Table 6:** Correlations between darts performance and ball-skill related items.

Pearson Correlation		Dart Score Short	CV Short	Dart Score Long	CV Long
All children n = 165	PERF-FIT				
	Bouncing and catching	0.36 **	−0.35 **	0.47 **	−0.45 **
	Throwing and catching	0.39 **	−0.39 **	0.52 **	−0.51 **
	Overhead Throw	0.38 **	−0.41 **	0.49 **	−0.51 **
	MABC-2				
	Catching	0.41 **	−0.36 **	0.49 **	−0.50 **
	Aiming	0.42 **	−0.34 **	0.46 **	−0.46 **

MABC-2 = Movement Assessment Battery for Children, CV = coefficient of variation. ** *p* < 0.001

**Table 7:** Results of the regression analysis for Model 2.

Dependent Variable	Predictor	Adjusted R^2^ in %	B	t	*p*	Model Fit
Throwing (MABC-2)	CV long-distance	26.9	−1.97	−2.77	0.006	F 31.2, *p* <0.001
	Score long-distance		0.60	2.43	0.016	
Aiming (MABC-2)	CV long-distance	22.9	−1.75	−2.49	0.015	F 25.35, *p* <0.001
	Score long-distance		0.55	2.23	0.027	
Bouncing and Catching (PERF-FIT)	Score long-distance	22.8	1.09	2.67	0.008	F 25.28, *p* <0.001
	CV long-distance		−2.38	−2.11	0.046	
Throwing and Catching(PERF-FIT)	Score long-distance	28.6	1.61	2.84	0.005	F 33.8, *p* <0.001
	CV long-distance		−4.24	−2.58	0.011	
Overhead throw (PERF-FIT)	CV long-distance	26.7	−27.57	−2.97	0.003	F 31.11, *p* <0.001
	Score long-distance		7.11	2.22	0.028	

MABC-2 = Movement Assessment Battery for Children, PERF-FIT = Performance and Fitness Test, CV = coefficient of variation, B: unstandardized value per variable, t; coefficient t-value. *p*: Calculated probability of each independent variable. Model fit: test statistics from the F-test with *p*-value.

## Data Availability

Data are available from the first author upon reasonable request.
